# Effect of Improving Spatial or Temporal Resolution on Image Quality and Quantitative Perfusion Assessment with *k-t* SENSE Acceleration in First-Pass CMR Myocardial Perfusion Imaging

**DOI:** 10.1002/mrm.22493

**Published:** 2010-09-27

**Authors:** Neil Maredia, Aleksandra Radjenovic, Sebastian Kozerke, Abdulghani Larghat, John P Greenwood, Sven Plein

**Affiliations:** 1 Division of Cardiovascular and Neuronal Remodelling, University of LeedsLeeds, United Kingdom; 2 School of Medicine, University of LeedsLeeds, United Kingdom; 3 Institute for Biomedical Engineering, University and ETH ZurichSwitzerland

**Keywords:** *k-t* SENSE, adenosine stress myocardial perfusion imaging, Fermi deconvolution, myocardial perfusion reserve index

## Abstract

*k-t* Sensitivity-encoded (*k-t* SENSE) acceleration has been used to improve spatial resolution, temporal resolution, and slice coverage in first-pass cardiac magnetic resonance myocardial perfusion imaging. This study compares the effect of investing the speed-up afforded by *k-t* SENSE acceleration in spatial or temporal resolution. Ten healthy volunteers underwent adenosine stress myocardial perfusion imaging using four saturation-recovery gradient echo perfusion sequences: a reference sequence accelerated by sensitivity encoding (SENSE), and three *k-t* SENSE–accelerated sequences with higher spatial resolution (“*k-t* High”), shorter acquisition window (“*k-t* Fast”), or a shared increase in both parameters (“*k-t* Hybrid”) relative to the reference. Dark-rim artifacts and image quality were analyzed. Semiquantitative myocardial perfusion reserve index (MPRI) and Fermi-derived quantitative MPR were also calculated. The *k-t* Hybrid sequence produced highest image quality scores at rest (*P* = 0.015). Rim artifact thickness and extent were lowest using *k-t* High and *k-t* Hybrid sequences (*P* < 0.001). There were no significant differences in MPRI and MPR values derived by each sequence. Maximizing spatial resolution by *k-t* SENSE acceleration produces the greatest reduction in dark rim artifact. There is good agreement between *k-t* SENSE and standard acquisition methods for semiquantitative and fully quantitative myocardial perfusion analysis. Magn Reson Med, 2010. © 2010 Wiley-Liss, Inc.

Dynamic first pass myocardial perfusion imaging by cardiac magnetic resonance (CMR) poses considerable challenges for pulse sequence design. To achieve adequate image resolution and slice coverage, large amounts of image data must be acquired in a short period of time. Parallel imaging techniques such as sensitivity encoding (SENSE) may be used to accelerate data acquisition, though signal-to-noise penalties limit SENSE acceleration factors to 2–3 when used for two-dimensional (2D) imaging (1).

Advanced acceleration techniques such as *k-t* broad-use linear acquisition speed-up technique (*k-t* BLAST) and *k-t* sensitivity encoding (*k-t* SENSE), exploit spatio-temporal correlations in dynamic imaging (2), permitting higher degrees of acceleration than SENSE, with fewer signal-to-noise penalties. The acceleration afforded by these techniques may be used to improve spatial resolution, reduce acquisition window duration, increase slice coverage, or a combination of these characteristics. Studies that have utilized *k-t* BLAST and *k-t* SENSE to accelerate CMR myocardial perfusion imaging to date have focused either on the optimization of acquisition window duration (3) or spatial resolution (4, 5). However, the lack of a direct comparison between these different approaches means the optimal mode of implementation, with regard to image quality, artifact reduction, and quantitative perfusion assessment, remains a matter of speculation.

To date, it is also unknown if data acquired with *k-t* SENSE can be used to estimate myocardial blood flow. The temporal undersampling, in particular, may lead to temporal blurring of data that may invalidate quantitative measurements. It is not known how different implementations of *k-t* SENSE affect such measurements.

This work aims to compare several potential ways in which to utilize the “speed-up” afforded by *k-t* SENSE in first-pass CMR myocardial perfusion imaging, and in so doing, investigate the effect of spatial and temporal resolution settings on dark rim artifact formation. In addition, the work investigates the potential for semiquantitative and fully quantitative assessment of myocardial perfusion using *k-t* SENSE accelerated CMR imaging in its different implementations.

## MATERIALS AND METHODS

### Subjects

Ten healthy volunteers, aged 31–60 years (mean 44 years; three males, seven females) were recruited by an e-mail sent to staff and students at the University of Leeds. Exclusion criteria for the study included contraindications to CMR (pacemakers, metallic implants, and claustrophobia) and adenosine (a history of reversible airways disease or advanced heart block), a history of cardiac disease, any other relevant medical condition or regular medication. The study design was approved by the Leeds (West) ethics committee and each subject gave written informed consent. Volunteers were asked to avoid caffeine for 12 hr preceding each scan.

### Scanner Hardware

The study was performed using a 1.5-Tesla Philips Intera MR imaging system (Philips, Best, The Netherlands), equipped with “Master” gradients. Maximum gradient strength and slew rate were 30 mT/m and 150 mT/m/ msec, respectively. A five-element cardiac phased-array synergy coil was used for signal reception during cardiac imaging. Vectorcardiographic (VCG) heart rate monitoring was performed to allow cardiac gating of image acquisition.

### Scan Protocols

Following survey and localizer scans, the stress perfusion study was planned in the short axis plane, parallel to the mitral valve annulus in orthogonal long axis views. Slices were positioned according to the “three of five” slice technique (6), ensuring maximal reproducibility between scans. Stress perfusion imaging commenced 4 min into an intravenous adenosine infusion, delivered at a rate of 140 mcg/kg/min. Breath-holding instructions varied according to the type of sequence used. For the SENSE-accelerated sequence, images were reviewed in real time during acquisition; hence the subjects were instructed to hold their breath when gadolinium entered the right ventricle and to breathe gently following completion of the first pass. As *k-t* SENSE images were not reviewable in real time, the subjects were instructed to hold their breath ∼2 sec after contrast injection, and advised to hold until acquisition was completed. Acquisition duration was set at 24 or 32 frames for the *k-t* SENSE sequences according to how well the volunteers performed during “dummy” perfusion runs. The SENSE acquisitions were of variable length, as they were stopped when the second pass of gadolinium through the left ventricular cavity was seen to occur but did not exceed 60 dynamics. Gadopentetate dimeglumine contrast (Magnevist®, Bayer, UK) was delivered intravenously at a dose of 0.05 mmol/kg and at a standard rate of 5 mL/sec, followed by a 15-mL saline flush delivered at the same rate. A 15-min rest period was observed to allow contrast to wash out of the myocardium. Rest perfusion imaging was then performed using identical settings as the stress perfusion scan.

At the subject's first visit, left ventricular function was also assessed using a multiple slice two-dimensional multiphase steady state free precession cine sequence covering the left ventricle (LV) in 10–12 short axis slices from apex to base.

### Pulse Sequence Design

The “speed-up” afforded by *k-t* SENSE can be applied to improve spatial resolution, temporal resolution, or myocardial slice coverage in CMR myocardial perfusion imaging. A three-slice imaging strategy is conventionally adopted in most centers as this allows assessment of basal, mid, and apical LV perfusion in accordance with the recommendations of the American Heart Association (7). Three-slice myocardial coverage was maintained throughout the study, permitting *k-t* SENSE acceleration to be applied to improve spatial and/or temporal resolution. To determine the optimal method of implementing *k-t* SENSE, subjects attended for stress and rest CMR myocardial perfusion imaging on four separate occasions.

The starting point for pulse sequence design was a conventional saturation-recovery spoiled gradient echo pulse sequence accelerated with twofold sensitivity encoding (SENSE) and producing an in-plane spatial resolution of 2.6 × 2.6 mm^2^. This pulse sequence is typical of those used in current clinical practice. Three *k-t* SENSE accelerated sequences were then created to largely mirror the SENSE sequence in as many parameters as possible, aside from the particular image attribute being tested (Table 1). The *k-t* High sequence had a similar acquisition window duration to the SENSE sequence but a superior spatial resolution. The *k-t* Fast sequence had an identical spatial resolution to the SENSE sequence but a shorter acquisition window duration. The *k-t* Hybrid sequence had a shorter acquisition window duration and higher spatial resolution relative to the standard SENSE sequence but was a compromise between these two parameters. To ensure that sequence designs differed by a sufficient margin, a *k-t* SENSE acceleration factor of 8 was adopted, together with half Fourier and partial echo techniques as required. “Training plug-in” was adopted for all *k-t* SENSE accelerated acquisitions (8).

**Table 1:** Pulse Sequence Characteristics

Sequence name	SENSE	*k-t* High	*k-t* Fast	*k-t* Hybrid
Acceleration method	SENSE	*k-t* SENSE	*k-t* SENSE	*k-t* SENSE
Acceleration factor	2	8	8	8
Training profiles	-	11	11	11
Partial echo	Yes	Yes	Yes	No
Half Fourier	Yes	No	No	No
Acquisition matrix	128	256	128	192
Reconstruction matrix	256	256	256	256
Acquired in-plane voxel dimensions (mm)^a^	2.66 × 2.66	1.33 × 1.33	2.66 × 2.76	1.77 × 1.82
Image acquisition time per slice (msec)	119	117	64	109
Number of dynamic frames acquired	Variable	24 or 32	24 or 32	24 or 32

a Assuming field of view size of 340 mm.

All other important pulse sequence characteristics were kept constant for each pulse sequence. A 90° saturation pulse was delivered to each slice and the delay between preparation pulse and image acquisition was 120 msec for each sequence. The shortest possible trigger delay was adopted for the first acquired slice and all subsequent slices were acquired with minimal possible delay from the preceding slice acquisition. Each sequence produced three 10-mm-thick myocardial slice images per dynamic frame. An 85% rectangular field of view was adopted for each sequence. The field of view size was altered according to volunteer size and kept constant for each of their four visits. Water fat shift values were constant at 0.35 pixels (bandwidth 620.3 Hz), the excitation angle was 15°, and for each pulse sequence, the shortest achievable repetition and echo times were selected.

### Image Analysis

Perfusion and volumetric images were analyzed using MASS version 6.1.6 (Medis, Leiden, The Netherlands). All image analysis was performed on the middle slice as this is least prone to partial volume effects or artifacts relating to the left ventricular outflow tract, more common in the apical and basal slices, respectively. The window settings of the viewing station were standardized for all image analysis. Image quality and breathing artifact scoring was performed by the consensus of two observers (NM and SP, 2 and 10 years experience in CMR myocardial perfusion imaging, respectively). Image quality was scored from 0 to 3: 0: poor, 1: reasonable, 2: good, 3: excellent. Breathing artifact was scored from 0 to 3: 0: none, 1: mild, 2: moderate, 3: severe.

Dark-rim artifact thickness was measured on the frame in which it appeared most prominent, using electronic calipers. The area of the artifact was assessed by contouring and expressed as a proportion of myocardial slice area. The duration of the artifact was recorded as the number of dynamic frames in which it was visible.

### Semiquantitative Assessment of Myocardial Perfusion

The epicardial and endocardial borders of the myocardium were contoured, excluding any suspected dark rim artifact from the region of interest, and the myocardium was divided into six segments. A region of interest was drawn in the left ventricular blood pool, avoiding any papillary muscles therein, to permit the derivation of an arterial input function. Contours were manually corrected for motion due to breathing or VCG mistriggering. Signal intensity/time curves were then drawn for each myocardial segment and the LV blood pool.

The maximal upslopes of the myocardial signal intensity curves were calculated using a five-point linear fit model. The maximal upslope of the LV blood pool curve was calculated using a three-point linear fit model, due to the shorter duration of the upslope relative to the myocardial slope. Mean myocardial upslopes (mean of the maximal upslopes for all segments in a myocardial slice) were corrected for the arterial input function and used to derive the myocardial perfusion reserve index (MPRI) (9, 10).

### Quantitative Myocardial Perfusion Assessment

Absolute myocardial blood flow values were estimated by Fermi-constrained deconvolution (11). Myocardial signal intensity data, generated as outlined above, were exported to a locally developed deconvolution program based on the Matlab software platform (version 7.7.0.471, R2008b, The Mathworks, Natick, MA). This software required manual input to equalize the baselines of the myocardial and LV blood pool curves, to identify the beginning and end of the first pass of contrast agent, and to correct the delay time between LV and myocardial blood pool curves, thereby allowing fitting to a Fermi function. The myocardial perfusion reserve (MPR) was calculated by dividing stress and rest myocardial blood flow values (11).

### Left Ventricular Function Assessment

Left ventricular function and mass parameters were derived from multiphase, multislice short axis cine images using conventional methods (12).

### Statistical Analysis

Statistical analysis was performed using SPSS version 15.0. All data were tested for normality using the Kolmogorov Smirnov test. Image quality scores were compared using Friedman's test for nonparametric repeated measures comparisons. Artifact thickness and extent, semiquantitative MPRI, and fully quantitative MPR values were compared using repeated measures analysis of variance (ANOVA) testing and Bonferroni correction was applied to pairwise comparisons.

The reproducibility of semiquantitative and fully quantitative assessments of myocardial perfusion in our unit has been published previously (13). The reproducibility of rim artifact thickness, duration, and extent measurements was assessed using the stress perfusion images from each volunteer study. To assess intraobserver variability, the original observer repeated his measurements 2 months after the original assessment. An additional observer (AL) independently measured image attributes to permit calculation of interobserver variability. The error between repeated measurements was assessed in the manner described by Bland and Altman (14) and was quoted in the form of within-subject standard deviation.

## RESULTS

**FIG. 1:**
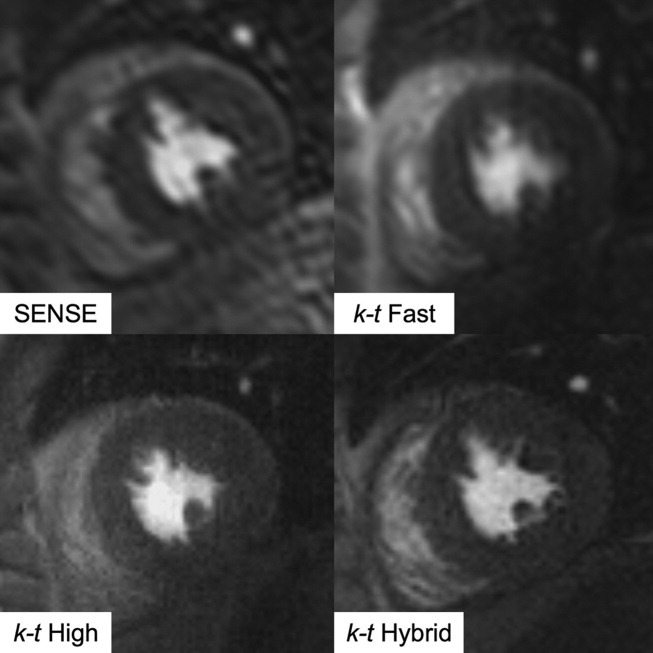
Resting myocardial perfusion images from a single volunteer.

In two of ten volunteers, equipment failure occurred during one of the four studies. Both scans were repeated during a fifth visit. Mean volunteer age was 44 (range 30–60), mean weight 72 kg (range 58–88kg), and median field of view 340 mm (range 320–360mm). All subjects had normal left ventricular systolic function (mean left ventricular ejection fraction 59%, range 57–62%). There was no significant difference in the heart rate achieved during vasodilator stress at visits 1–4, as measured after 2 min of intravenous adenosine (mean heart rates 94, 89, 93, and 92 bpm respectively; *P* = 0.54). No significant adverse events occurred during adenosine infusion. Sample images from a single volunteer, using the four sequence types, may be seen in Fig. 1.

### Image Attributes

Image quality scores during stress were similar for all sequence types (Table 2). At rest however, the *k-t* Hybrid sequence produced significantly higher image quality than the other three sequence types.

**Table 2:** Mid-Slice Image Quality and Rim-Artifact Characteristics (*n* = 10)

		SENSE	*k-t* High	*k-t* Fast	*k-t* Hybrid	Four sequence comparison *P* value^a^	Three *k-t* sequence comparison *P* value^b^
Mean image quality score	Stress	1.7	1.6	1.7	1.9	0.590	0.325
	Rest	2	2.1	1.9	2.4	0.015	0.022
Mean rim thickness (mm)	Stress	3.4	1.1	3.0	1.8	<0.001	<0.001
	Rest	3.4	1.3	2.7	2.2	<0.001	<0.001
Mean rim extent (%)	Stress	16.1	2.2	6.0	4.6	<0.001	<0.001
	Rest	14.5	1.7	5.0	4.7	<0.001	0.002
Mean rim duration (frames)	Stress	11	7	11	10	0.07	0.06
	Rest	13	9	11	13	0.008	0.07

a *P* value for statistical comparison of all four sequences.

b *P* value for statistical comparison of the three *k-t* SENSE accelerated sequences.

There were significant differences in rim-artifact thickness and rim-artifact extent between the four sequences analyzed together and the three *k-t* SENSE sequences analyzed in isolation. Tables 3 and 4 show the results of pairwise ANOVA comparisons using Bonferroni correction. SENSE and *k-t* Fast perfusion images demonstrated rim artifacts of similar thickness at both stress and rest. Both of these sequence types produced significantly thicker rim artifacts than *k-t* High and *k-t* Hybrid types, at both stress and rest. There was no significant difference in rim artifact thickness between *k-t* High and *k-t* Hybrid types in either physiological state. Myocardial rim artifact extent was significantly larger in the SENSE images than any other image type. *k-t* Fast images demonstrated similar artifact extent to *k-t* Hybrid images but significantly more than the *k-t* High images. At rest, *k-t* High images contained significantly smaller artifact than *k-t* Hybrid images though only a trend toward significance was seen in the corresponding images during stress.

**Table 3:** Comparison of Rim Artifact Thickness Between Pulse Sequences

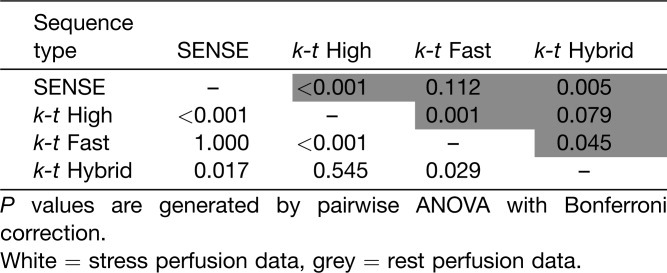

**Table 4:** Comparison of Rim Artifact Extent Between Pulse Sequences

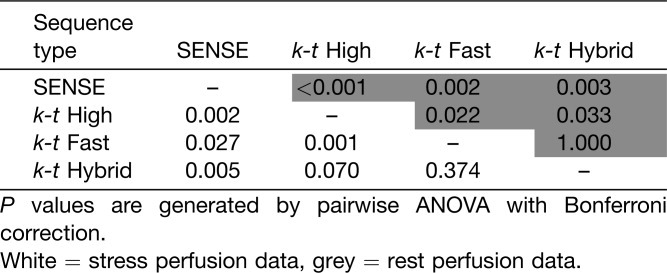

There was no significant difference in rim-artifact duration between the four sequence types during stress. A four-sequence comparison of rest images suggested significant differences in rim-artifact duration but pairwise comparisons demonstrated no difference between individual sequence types in either physiological state.

**FIG. 2:**
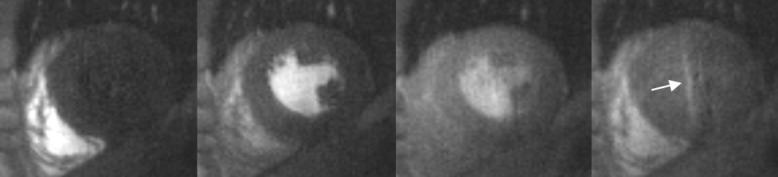
Respiratory artifact occurring toward the end of a *k-t* High stress perfusion study.

Breathing-related artifacts were found in 57% of all *k-t* accelerated stress perfusion studies and 17% of rest perfusion studies (*P* = 0.052) generally occurring toward the end of a breathhold, as demonstrated in Fig. 2. During stress, 70% of *k-t* High and 50% of both *k-t* Fast and *k-t* Hybrid images showed respiratory artifacts (*P* = 0.52). At rest, 30% of *k-t* High, 20% of *k-t* Hybrid, and no *k-t* Fast images demonstrated respiratory artifacts (*P* = 0.1). SENSE accelerated scans demonstrated no breathing-related artifacts.

### Reproducibility

The interobserver and intraobserver variability of artifact measurements was generally low (Table 5).

**Table 5:** Interobserver and Intraobserver Reproducibility

		Within-subject standard deviation	
		
Sequence type	Image attribute	Interobserver	Intraobserver
SENSE	Rim duration (frames)	1.26	0.50
	Rim thickness (mm)	0.26	0.29
	Rim extent (%)	1.87	1.69
*k-t* High	Rim duration (frames)	0.95	0.67
	Rim thickness (mm)	0.32	0.26
	Rim extent (%)	0.59	0.76
*k-t* Fast	Rim duration (frames)	1.87	1.05
	Rim thickness (mm)	0.31	0.20
	Rim extent (%)	1.79	1.24
*k-t* Hybrid	Rim duration (frames)	1.32	0.92
	Rim thickness (mm)	0.30	0.21
	Rim extent (%)	0.83	0.41

Stress perfusion data from 10 volunteers.

### Estimates of Myocardial Perfusion

**FIG. 3:**
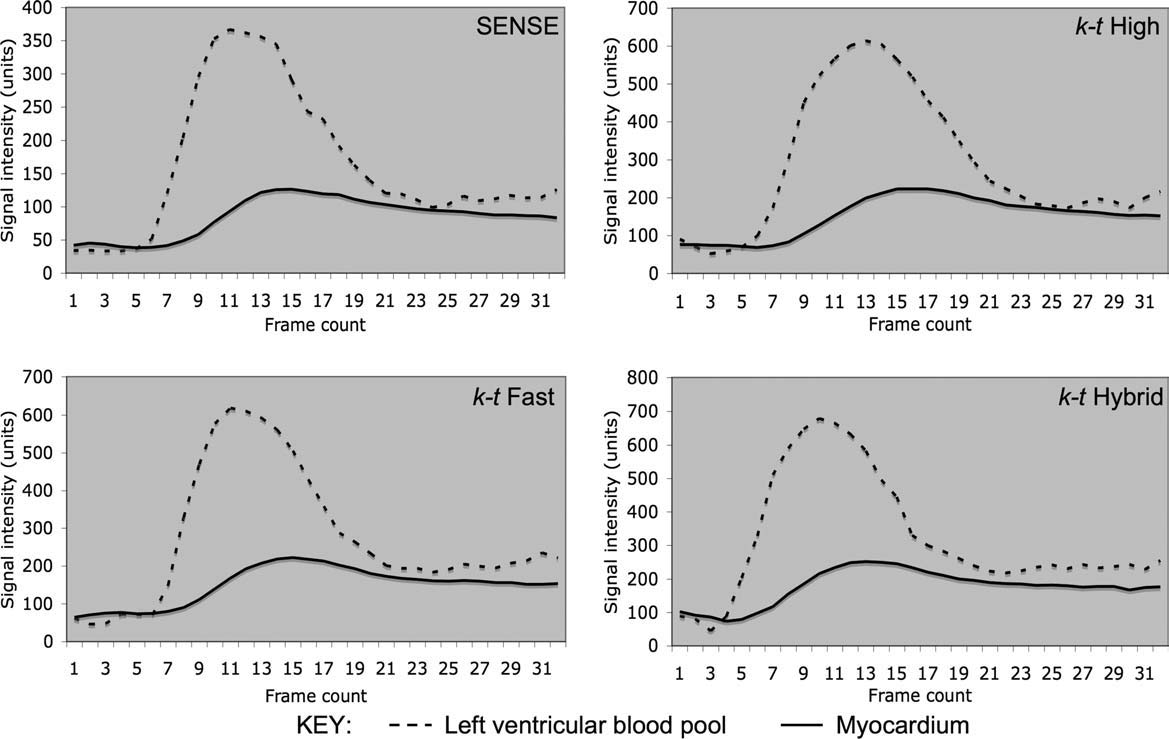
Myocardial perfusion signal intensity–time curves obtained using each sequence type during adenosine stress (all curves are from the same volunteer).

Signal intensity–time curves obtained using the four sequence types on a single volunteer during adenosine stress are shown in Fig. 3. Although there are differences in baseline and peak signal intensities (measured in arbitrary units) between the four acquisitions reflecting the fact they were acquired on four different occasions, the relative change in signal intensity, and the overall shape of the curves were similar between sequences. There was no appreciable temporal smoothing with any of the three *k*-*t* accelerated sequences.

### Semiquantitative Assessment

It was not possible to accurately measure myocardial upslopes from the *k-t* Hybrid images for three volunteers due to a combination of technical failures during scanning and incorrect timing of image acquisition after contrast delivery. These three volunteers (numbers 4, 6, and 7) were therefore excluded from the analysis.

**FIG. 4:**
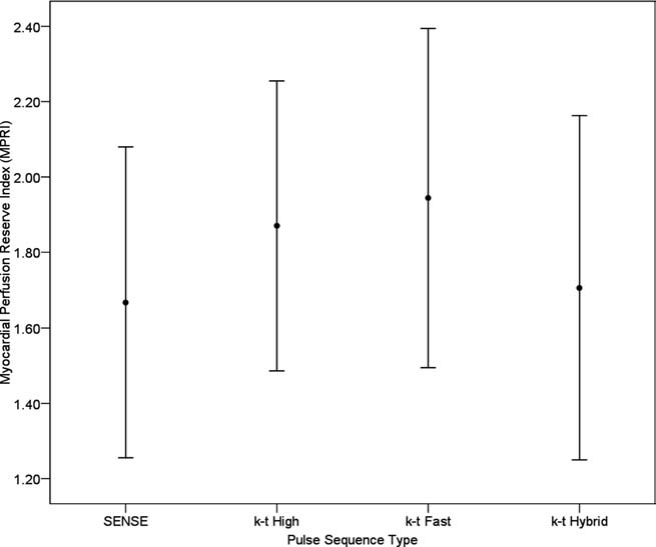
Mean mid-slice myocardial perfusion reserve index (MPRI) results. (Error bars indicate ± one standard deviation).

Mean mid-slice MPRI values in the remaining seven volunteers for the SENSE sequence were 1.67, *k-t* High 1.87, *k-t* Fast 1.94, and *k-t* Hybrid 1.71 (Fig. 4). There was no significant difference in the mean mid-slice MPRI values derived from each sequence type (*P* = 0.265).

### Quantitative Perfusion Assessment by Fermi Deconvolution

Volunteers 4, 7, and 9 were excluded from this analysis due to late commencement of image acquisition after contrast delivery, making it impossible to accurately set the baseline signal intensity in the LV blood pool when performing deconvolution.

**FIG. 5:**
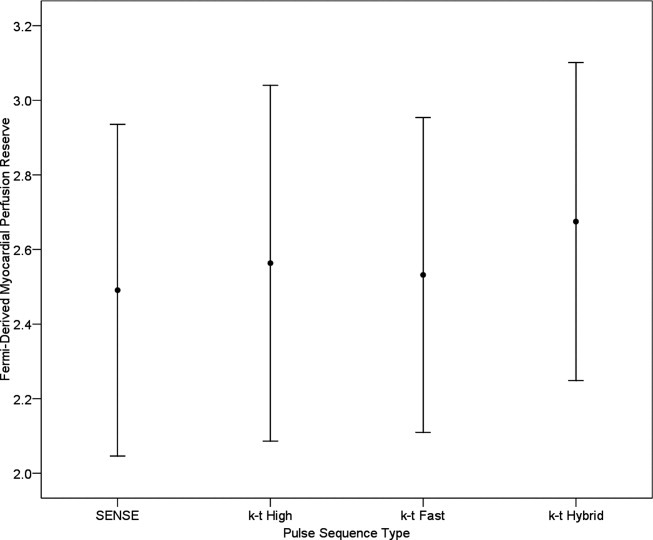
Mean mid-slice Fermi deconvolution–derived myocardial perfusion reserve (MPR) results. (Error bars indicate ± one standard deviation).

Mean mid-slice MPR for the SENSE sequence was 2.49, *k-t* High 2.56, *k-t* Fast 2.53, and *k-t* Hybrid 2.67 (*P* = 0.58; Fig. 5 and Table 6). Stress and rest absolute myocardial blood flow values did not differ significantly between sequence types (*P* = 0.33 and 0.28, respectively).

**Table 6:** Absolute Myocardial Blood Flow and Myocardial Perfusion Reserve Results (*n* = 7)

	SENSE	*k-t* High	*k-t* Fast	*k-t* Hybrid	*P* value
Mean stress blood flow (ml/min/g)	4.24	3.88	4.54	4.13	0.33
Mean rest blood flow (ml/min/g)	1.79	1.57	1.82	1.56	0.28
Mean myocardial perfusion reserve	2.49	2.56	2.53	2.67	0.58

## DISCUSSION

This study provides first evidence as to how the accelerated data acquisition afforded by spatio-temporal undersampling methods such as *k-t* SENSE may be utilized in CMR first pass myocardial perfusion. We found that dark-rim artifacts can be minimized by utilizing *k-t* SENSE acceleration to improve spatial resolution. In addition, we have shown for the first time that myocardial perfusion data acquired with *k-t* SENSE yield similar quantitative estimates of myocardial perfusion to a conventional acquisition method.

Several methods have recently been proposed that permit signal-to-noise ratio–efficient acceleration of data for dynamic and cine CMR applications, including *k-t* SENSE, *k-t* BLAST, T-SENSE, and others (2, 15, 16). These methods offer new opportunities for contrast-enhanced myocardial perfusion CMR, where dense data sampling is required and pulse sequence design is driven by compromises between spatial resolution, acquisition window duration, myocardial coverage, and image quality. The speed up provided by the new acceleration methods can be invested flexibly into any of these variables and previous studies have used them in different ways. Some of the effects of changing resolution and other acquisition parameters may be modeled or tested in phantoms (e.g., effect on signal to noise or contrast to noise ratio). Others, such as image quality, temporal contrast behavior, or performance under pharmacological stress conditions are more complex and require in vivo testing. Comparative studies in vivo, however, are challenging and the choice of parameters that can be compared is limited by the logistics of in vivo studies. In this study, we selected two of the most likely targets for accelerated data acquisition in CMR myocardial perfusion imaging—spatial resolution and acquisition window duration. It is acknowledged that the choices we made are arbitrary, but we were able to derive several generalizable observations from our data.

Overall image quality scores were significantly different between the three implementations of *k-t* SENSE, although absolute differences were small. When compared with standard SENSE-accelerated acquisition, image quality was also largely similar. Although individual images were often perceived to be of higher quality using the hybrid and high spatial resolution implementations of *k-t* SENSE, respiratory motion artifacts (which did not occur with the SENSE accelerated sequence) often had a negative effect on image quality. However, such respiratory artifacts generally occur towards the end of an acquisition, when breath holding became less consistent (5). Consequently, there would be little impact on the Fermi-constrained quantitative assessment which fits the signal-response function of the first pass of the time series only.

We found significant differences in the presence and extent of subendocardial dark-rim artifacts. These artifacts are a frequent finding in CMR myocardial perfusion imaging and may create diagnostic uncertainty especially for less-experienced CMR observers because of the potential to mimic genuine perfusion defects. A number of factors have been attributed to this phenomenon, including magnetic susceptibility, cardiac motion during image acquisition, and Gibbs ringing (which itself relates to spatial resolution) (17). In an earlier study, a high spatial resolution implementation of *k-t* SENSE for CMR myocardial perfusion imaging produced a reduction in dark rim artifact when compared with standard SENSE accelerated imaging (4). Phantom studies using SENSE acceleration have also shown that reductions in image acquisition time led to smaller rim artifacts (18). However, when acquisition window duration was reduced in a study of CMR myocardial perfusion imaging accelerated by *k-t* BLAST, the effect on dark rim artifact size was not reported (3). By directly comparing these different strategies, our study aims to inform the debate as to how best to implement advanced acceleration techniques (such as *k-t* SENSE) in CMR myocardial perfusion imaging.

In our study, it has been shown that dark-rim artifact thickness is significantly affected by the spatial resolution of the pulse sequence, whereas alterations in acquisition window duration did not produce such an effect. However, the size of the artifact, when measured as a proportion of the total myocardial area in a slice, is influenced by both acquisition window and spatial resolution parameters. The largest artifact extent was seen with the SENSE accelerated sequence, and although improvements were achieved by reductions in acquisition window duration, increases in spatial resolution and a combination of the two, the greatest reduction in artifact extent was again seen with the high spatial resolution sequence.

A previous study has shown that SENSE and *k-t* SENSE derived MPRI values did not differ significantly (4). The present study has now demonstrated that *k-t* SENSE derived estimates of absolute myocardial blood flow are also consistent with measurements based on conventional acquisition. Furthermore, the MPRI values in this study are consistent with those published previously (19, 20). Given the high temporal undersampling that takes place in *k-t* SENSE acquisition (eightfold in the present study), this evidence of temporal fidelity in the acquired data provides important reassurance. By using advanced acceleration techniques to boost spatial resolution, it may become possible to quantitatively analyze transmural perfusion gradients with greater accuracy than before.

### Limitations

To produce pulse sequence designs with the desired characteristics, some sequence designs included partial echo and partial Fourier techniques, whereas others did not. Our reconstructions incorporated a finite impulse response (FIR) filter approach (21). The performance of the partial Fourier algorithm is dependent on the underlying image phase estimate and if this estimate is correct, the reconstructed image is very close to the one acquired without partial Fourier (except for noise). As the echo times are very short in the perfusion sequences used, the phase variation across the image is relatively benign and phase estimation should be robust. Consequently, little difference would be expected between partial Fourier and nonpartial Fourier reconstructions. It, therefore, appears unlikely that our implementation of these techniques would have a significant impact on the results of our study. Temporal filtering arising during *k-t* SENSE reconstruction may also have played a role in reducing dark rim artifacts by potentially reducing peak signal intensity in the left ventricle. However, the magnitude of this effect on dark rim artifact size is expected to be significantly smaller than spatial resolution considerations, which play a key role in determining the extent of dark banding artifacts.

A “shortest” trigger delay was adopted to permit three slice acquisitions during every R-R interval, even at high heart rates. Consequently, the point in the cardiac cycle at which slices 2 and 3 were imaged will have varied between sequences where the acquisition window per slice was different. This could, in turn, lead to varying degrees of cardiac motion at the time of image acquisition, which may have an impact on artifact size. Although trigger delays may be prespecified in an attempt to acquire images during periods of relatively reduced cardiac motion, there is significant variation in rest periods between different individuals, and in different physiological states (rest/stress). We rejected this strategy as it would compromise our ability to acquire three slice images per R-R interval at the higher heart rates encountered during adenosine stress. It should, however, be noted that delay times are measured to the center of *k*-space so any difference in timings would be expected to be small.

As all of our subjects were healthy volunteers, it was not possible to assess the effect of sequence parameter alterations on diagnostic accuracy. It is hypothesized that reductions in artifact size may permit an improvement in the detection of genuine ischemia by CMR, but performing studies with multiple comparisons in large patient populations is challenging.

It was necessary to exclude three volunteers from the semiquantitative and fully quantitative analyses due to technical issues (most commonly, incorrect timing of image acquisition relative to the first pass of contrast through the LV). Because of the spatiotemporal correlations inherent to *k-t* SENSE, and unlike standard SENSE acceleration, it is not possible to view image acquisition in real-time. *k-t* SENSE–accelerated scans are, therefore, usually kept to a short breath-hold to minimize artifacts, making correct timing of the acquisition more challenging. Further developments of *k-t* SENSE may reduce sensitivity to respiratory motion, permitting longer acquisitions and thereby reducing the potential for inaccuracies in timing of image acquisition relative to contrast delivery.

The semiquantitative and fully quantitative assessments of myocardial perfusion undertaken in this study were made globally over the entire myocardial circumference, rather than on a segmental basis. Further work would be needed to establish whether segmental analysis is sufficiently accurate using *k-t* SENSE to allow comparison of blood flows between different coronary artery territories. Furthermore, relatively large standard deviations in MPR and MPRI values were found with each sequence type, which in the context of small sample sizes, might have made it difficult to detect differences if they did actually exist.

## CONCLUSION

Dark-rim artifacts may be minimized by utilizing *k-t* SENSE acceleration to maximize spatial resolution in CMR myocardial perfusion imaging. When the speed-up offered by *k-t* SENSE was targeted at reducing acquisition window duration or combined between acquisition window and spatial resolution, some improvement in artifact extent was seen, though to a lesser extent than when spatial resolution was improved alone. Semiquantitative and fully quantitative measures of MPR and MPRI were found to be comparable between *k-t* SENSE and SENSE acceleration techniques. The current implementation of *k-t* SENSE acceleration remains sensitive to respiratory motion, particularly during stress, which limits scan duration to a single breathhold.
